# LB1. Remdesivir for the Treatment of High-Risk Non-Hospitalized Individuals With COVID-19: A Randomized, Double-Blind, Placebo-Controlled Trial

**DOI:** 10.1093/ofid/ofab466.1642

**Published:** 2021-12-04

**Authors:** Joshua A Hill, Roger Paredes, Carlos Vaca, Jorge Mera, Brandon J Webb, Gilberto Perez, Godson Oguchi, Pablo Ryan, Jan Gerstoft, Michael Brown, Joshua Schiffer, Samuel Brown, Morgan Katz, Adit A Ginde, Gregory Camus, Danielle P Porter, Robert H Hyland, Shuguang Chen, Kavita Juneja, Anu Osinusi, Frank Duff, Robert L Gottlieb

**Affiliations:** 1 Fred Hutchinson Cancer Research Center; University of Washington, Seattle, WA; 2 Hospital Universitario Germans Trias i Pujol, Badalona, Catalonia, Spain; 3 Nuren Medical and Research Center, Miami, Florida; 4 Cherokee Nation Outpatient Health Center, Tahlequah, Oklahoma; 5 Intermountain Healthcare, Murray, UT; 6 Evolution Clinical Trials, Hialeah Gardens, Florida; 7 Midland Florida Clinical Research Center, DeLand, Florida; 8 Hospital Universitario Infanta Leonor, Universidad Complutense de Madrid, Madrid, Madrid, Spain; 9 University of Copenhagen, Copenhagen, Hovedstaden, Denmark; 10 University College London Hospitals NHS Foundation Trust, London, England, United Kingdom; 11 Fred Hutch, Seattle, Washington; 12 Intermountain Medical Center, Murray, Utah; 13 Johns Hopkins University, Baltimore, MD; 14 University of Colorado, Aurora, Colorado; 15 Gilead Sciences, Inc, Foster City, California; 16 Gilead Sciences, Inc., Foster City, California; 17 AlloVir, Chapel Hill, North Carolina; 18 Gilead, Foster City, California; 19 Baylor University Medical Center, Dallas, Texas

## Abstract

**Background:**

Remdesivir (RDV) is a potent nucleotide prodrug inhibitor of the SARS-CoV-2 RNA-dependent RNA polymerase that has demonstrated efficacy in the treatment of patients hospitalized with moderate to severe COVID-19. This Phase 3 (GS-US-540–9012) double-blind, placebo-controlled study compared the efficacy and safety of 3 days of RDV to standard of care in non-hospitalized, high-risk participants with confirmed COVID-19.

Table 1. COVID-19 related hospitalization or death, COVID-19 related medically attended visits or death, and Treatment Emergent Adverse Events

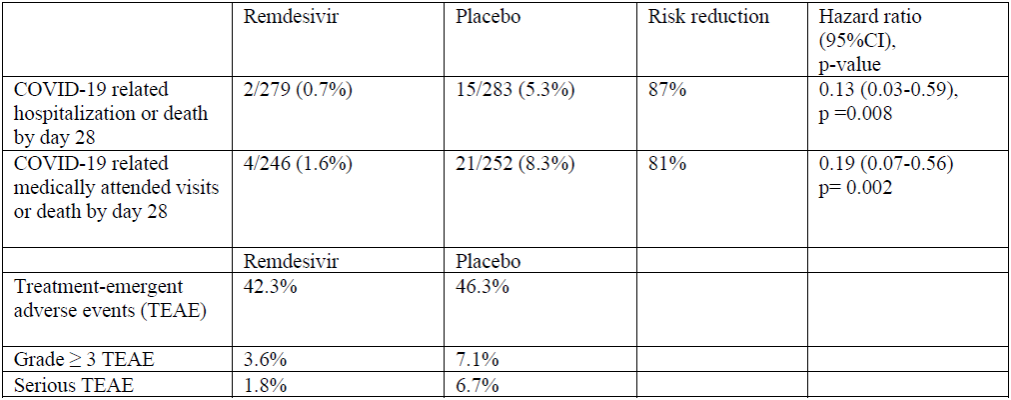

**Methods:**

Participants were randomly assigned 1:1 to receive intravenous (IV) RDV (200 mg on day 1, 100 mg on days 2 to 3) or placebo. The primary efficacy endpoint was composite COVID-19 hospitalization or all-cause death by day 28 and compared using Cox proportional hazards model with baseline stratification factors as covariates. The primary safety endpoint was proportion of participants with treatment-emergent adverse events. Study enrollment was terminated early for administrative reasons in light of the evolving pandemic.

**Results:**

562 patients underwent randomization and started their assigned treatment (279, RDV; 283, placebo). Baseline demographics and characteristics were balanced across arms. Overall, 52% were male, 44% were Hispanic/Latino ethnicity and 30% were ≥ 60 years old. The most common comorbidities were diabetes mellitus (62%), obesity (56%; median BMI, 30.7), and hypertension (48%). Median baseline SARS-CoV-2 RNA nasopharyngeal viral load was 6.2 log_10_ copies/mL. Treatment with RDV significantly reduced COVID-19 hospitalization or all-cause death by day 28 (HR, 0.13; 95% CI, 0.03 – 0.59; p = 0.008; Table 1) compared to placebo. Participants receiving RDV also had significantly lower risk for COVID-19-related medically attended visits or all-cause death by day 28 compared to placebo (HR, 0.19; 95% CI, 0.07 – 0.56; p = 0.002; Table 1). No deaths occurred in either arm by day 28. There was no difference between arms in time-weighted average change in nasopharyngeal viral loads from baseline up to day 7. The proportion of patients with AEs was similar between arms (Table 1); the most common AEs in the RDV arm were nausea (11%), headache (6%), and diarrhea (4%).

**Conclusion:**

A 3-day course of IV RDV was safe, well tolerated and highly effective at preventing COVID-19 related hospitalization or death in high-risk non-hospitalized COVID-19 patients.

**Disclosures:**

**Joshua A. Hill, MD**, Allogene (Individual(s) Involved: Self): Consultant; Allovir (Individual(s) Involved: Self): Consultant, Grant/Research Support; Amplyx (Individual(s) Involved: Self): Consultant; Covance/CSL (Individual(s) Involved: Self): Consultant; CRISPR (Individual(s) Involved: Self): Consultant; Gilead (Individual(s) Involved: Self): Consultant, Grant/Research Support; Karius: Grant/Research Support, Scientific Research Study Investigator; Medscape (Individual(s) Involved: Self): Consultant; Octapharma (Individual(s) Involved: Self): Consultant; OptumHealth (Individual(s) Involved: Self): Consultant; Takeda (Individual(s) Involved: Self): Consultant, Grant/Research Support, Scientific Research Study Investigator **Roger Paredes, MD, PhD**, **Gilead Sciences, Inc** (Grant/Research Support, Scientific Research Study Investigator, Advisor or Review Panel member) **Carlos Vaca, MD**, **Gilead Sciences, Inc** (Scientific Research Study Investigator) **Jorge Mera, MD**, **Gilead Sciences, Inc** (Consultant, Study Investigator (payment to employer not self)) **Gilberto Perez, MD**, **Gilead Sciences, Inc** (Scientific Research Study Investigator) **Godson Oguchi, MD**, **Gilead Sciences, Inc** (Scientific Research Study Investigator) **Pablo Ryan, MD PhD**, **Gilead Sciences, Inc** (Grant/Research Support, Scientific Research Study Investigator, Advisor or Review Panel member) **Jan Gerstoft, MD**, **Gilead Sciences, Inc** (Other Financial or Material Support, Study Investigator (payment to employer)) **Michael Brown, FRCP PhD**, **Gilead Sciences, Inc** (Scientific Research Study Investigator, Investigator for numerous remdesivir trials (employer received compensation)) **Morgan Katz, MD, MHS**, Roche (Individual(s) Involved: Self): Advisor or Review Panel member; Skinclique (Individual(s) Involved: Self): Consultant **Gregory Camus, PhD**, **Gilead Sciences** (Employee, Shareholder) **Danielle P. Porter, PhD**, **Gilead Sciences** (Employee, Shareholder) **Robert H. Hyland, DPhil**, **Gilead Sciences, Inc** (Shareholder, Other Financial or Material Support, Employee during the conduct of this trial) **Shuguang Chen, PhD**, **Gilead Sciences, Inc** (Employee, Shareholder) **Kavita Juneja, MD**, **Gilead Sciences, Inc** (Employee) **Anu Osinusi, MD**, **Gilead Sciences, Inc** (Employee, Shareholder) **Frank Duff, MD**, **Gilead Sciences, Inc** (Employee, Shareholder) **Robert L. Gottlieb, MD**, **Eli Lilly** (Scientific Research Study Investigator, Advisor or Review Panel member)**Gilead Sciences** (Scientific Research Study Investigator, Advisor or Review Panel member, Other Financial or Material Support, Gift in kind to Baylor Scott and White Research Institute for NCT03383419)**GSK** (Advisor or Review Panel member)**Johnson and Johnson** (Scientific Research Study Investigator)**Kinevant** (Scientific Research Study Investigator)**Roche/Genentech** (Scientific Research Study Investigator)

